# Identification and expression profiles of the YABBY transcription factors in wheat

**DOI:** 10.7717/peerj.12855

**Published:** 2022-02-03

**Authors:** Lidong Hao, Jinshan Zhang, Shubing Shi, Peng Li, Dandan Li, Tianjiao Zhang, Haibin Guo

**Affiliations:** 1 Xinjiang Agricultural University, College of Agriculture, Urumqi, Xinjiang, China; 2 Suihua University, College of Agriculture and Hydraulic Engineering, Suihua, Heilongjiang, China

**Keywords:** YABBY, Wheat, Genome-wide, Salt, Expression patterns

## Abstract

**Background:**

YABBY is a plant-specific transcription factor (TF) that belongs to the zinc finger protein superfamily and is composed of a C2–C2 domain at the N-terminus and a YABBY domain at the C-terminus. It plays a role in plant development and growth.

**Methods:**

In this study, 20 YABBY TFs were identified in the wheat genome. Phylogenetic relationships, collinearity relationships, gene structures, conserved motifs, and expression patterns were analyzed.

**Results:**

Twenty TaYABBY TFs were distributed unevenly on 15 chromosomes. Collinearity analysis showed that these genes have a close relationship with monocot plants. The phylogenetic tree of wheat YABBYs classified these TaYABBYs into FIL, YAB2, INO, and CRC clades. Gene structure and conserved motif analyses showed that they share similar components in the same clades. Expression profile analysis showed that many TaYABBY genes have high expression levels in leaf tissues and are regulated by abiotic stresses, especially salt stress. Our results provide a basis for further functional characterization of the YABBY gene family.

## Introduction

YABBY is a plant-specific transcription factor (TF) that is characterized by a zinc finger-like domain (C_2_–C_2_) in the N-terminus and a helix-loop-helix domain at the C-terminus region (Eckardt, 2010). There are six YABBY members in *Arabidopsis* are : *FILAMENTOUS FLOWER* (*FIL*), *YABBY3* (*YAB3*), *CRABS CLAW* (*CRC*), *INNER NO OUTER* (INO), *YABBY2* (*YAB2*), and *YABBY5* (*YAB5*) (Sarojam et al., 2010; Siegfried et al., 1999). *CRC* and *INO* are considered as “reproductive-specific genes”; while *YAB2*, *YAB3*, *YAB5*, and *FIL* are “vegetative genes”, they function redundantly to promote the development of a lateral organ (Sarojam et al., 2010).

Studies have found that *YABBY* genes function in plant development and growth. The FIL member *OsYABBY4* is predominantly expressed in the vascular tissues of rice and regulates vascular development (Yang, Ma & Li, 2016), while rice YAB2 member *OsYABBY1*, maize FIL/YAB3 members *ZYB9* and *ZYB14*, and *Arabidopsis YAB2*, *YAB3*, and *YAB5* have redundant functions that promote lateral organ development (Juarez, Twigg & Timmermans, 2004; Siegfried et al., 1999). *CRC* is essential for establishing polarity in the development of carpels and nectaries (Villanueva et al., 1999), *ZmYABBY1* and *ZmYABBY11* regulate male floret development (Strable & Vollbrecht, 2019), and rice *DROOPING LEAF* regulates the development of rice floral carpels and the formation of the leaf midrib (Ohmori et al., 2011; Yamaguchi et al., 2004). Rice and maize CRC members also have a conserved function in leaf development, affecting leaf width and length, leaf angle, and internode diameter (Nagasawa et al., 2003; Ohmori et al., 2011; Strable et al., 2017). *INO* promotes the development of the ovule exoderm into the seed coat (Villanueva et al., 1999). INO member *Arabidopsis INNER-NO-OUTER* is involved in epicarp formation and development (Simon et al., 2017).

*YABBY* genes also participate in responses to phytohormone responses. For example, overexpression of rice *OsYABBY1* results in a semi-dwarf phenotype by feedback regulation of gibberellin (GA) biosynthesis and metabolism (Toriba et al., 2007); *OsYAB4* regulates plant development and growth by regulating the GA signalling pathway (Yang, Ma & Li, 2016). *YABBY* genes are also involved in abiotic stress. For example, overexpression of pineapple *AcYABBY4* in Arabidopsis negatively regulates salt resistance in plants (Li & Li, 2019). Genome-wide analysis of *Phaseolus vulgaris* YABBY genes revealed that they are involved in salt stress (İnal et al., 2017), and soybean gene *GmYABBY10* negatively regulates drought and salt tolerance in plants (Zhao et al., 2017).

To date, identification of YABBY TFs has been performed in different plant species through genome-wide analyses. A total of six YABBY TFs have been identified in Arabidopsis (Siegfried et al., 1999), 17 in soybean (*Glycine max*) (Zhao et al., 2017), 9 in pineapple *(Ananas comosus)* (Li & Li, 2019), seven in grapevine (*Vitis vinifera*) (Zhang et al., 2019), nine in tomato *(Solanum lycopersicum)* (Huang et al., 2013), 12 in *Gossypium arboreum*, 12 in *G. raimondii*, 23 in *G. hirsutum* (Yang et al., 2018), and 16 in moso bamboo (*Phyllostachys edulis)* (Ma et al., 2021). As one of the most important crops worldwide, the genome of wheat has been sequenced; however, few studies have been conducted on the wheat *YABBY* gene family. This study aimed to carry out a comprehensive analysis of on the phylogenetic relationship, segmental duplication, chromosome location by *in silico* and expression profiling of wheat YABBY genes by qRT-PCR. Our study lays a foundation for future understanding of the evolution and function of wheat *YABBY* genes.

## Materials and Methods

### The identification of YABBY TFs

The coding sequence, protein sequence, and genome sequence of wheat (IWGSC), rice, and *Arabidopsis* were downloaded from Ensembl Plants (http://plants.ensembl.org/index.html). To identify wheat *YABBY* TFs, four steps were performed. First, we compared the *Arabidopsis* YABBY protein sequences against the wheat genome protein sequences using BLASTP. Second, we used PF04690, a characteristic profile of YABBY TFs from the PFAM database, to run HMMsearch against the wheat protein database with the threshold E< e^−5^. Third, we combined the results from the two steps above and manually removed the redundancy and alternative splicing genes. Fourth, the protein sequences of YABBY were submitted to NCBI’s Conserved Domain Database (NCBI CDD) and proteins without YABBY domains were also removed to confirm whether the putative YABBY protein contained the YABBY domain. Finally, putative wheat YABBY TFs were identified.

The physicochemical properties of wheat YABBYs were predicted by using ExPASy (Wilkins et al., 1999), and the amino acid number, theoretical isoelectric point (pI), molecular weight (MW), and grand average of hydropathicity (GRAVY) were predicted using ExPASy’s ProtParam tool (Wilkins et al., 1999). The subcellular location of TaYABBYs was predicted using CELLO v.2.5 (Yu et al., 2006) and Plant-mPLoc (Chou & Shen, 2010).

### Phylogenetic relationship, gene structure, and conserved motif analyses

Sequence alignments of YABBY proteins were generated using ClustalW. An unrooted neighbour-joining (NJ) phylogenetic tree was constructed using MEGA7 (Kumar, Stecher & Tamura, 2016) with 1,000 replicates based on aligning the full-length YABBY protein sequence from rice, *Arabidopsis*, and wheat. To further validate the accuracy of the NJ tree, an unrooted maximum likelihood (ML) tree was built using MEGA7 (Kumar, Stecher & Tamura, 2016) with 1,000 replicates. The gene structure of *TaYABBY* genes was constructed using the Gene Structure Display Server (GSDS 2.0) (Hu et al., 2015). The conserved motifs of TaYABBYs were predicted using MEME suite (Bailey et al., 2015) with the following parameters: maximum number = 10, motif width = 6–100 amino acids.

### Chromosomal location and collinearity analysis

All wheat *YABBY* genes were mapped onto the wheat chromosomes according to the information obtained from the Ensembl Plants database. The MCScanX program (Wang et al., 2012) was used to predict collinearity relationships between wheat and other species. The chromosomal location and collinearity relationship were visualised by TBtools (Chen et al., 2020).

### Plant materials, RNA isolation, cDNA synthesis, and quantitative RT- PCR

The wheat cultivar Chinese Spring (*Triticum aestivum*) was used in this study. For different tissue expression analyses, the roots, stems, leaves, and inflorescences were collected at the spike formation stage. For different abiotic stresses, 7-day-old seedlings were subjected to salt (200 mM NaCl), drought (20% polyethylene glycol [PEG] 6000), heat (42 °C), and cold (4 °C) for 2h in hydroponic culture to obtain whole plants and collected for RNA isolation. For salt stress at different time points, 7-day-old seedlings were subjected to 200 mM NaCl and collected at 0, 1, 2, 3, 5, 7, 12, and 24 h. Three replicates were performed per treatment, and each replicate included at least 15 plants. After collection, the samples were stored at −80 °C. Total RNA was isolated using the TRIzol reagent (TIANGEN Biotech, Beijing, China) and treated with RNase-free DNase I according the manufacturer’s instruction. The first-strand cDNA synthesis was performed according to the manufacturer’s instructions (TIANGEN Biotech, Beijing, China). Quantitative real-time PCR analysis was performed using Thermo Fish Q3 (Thermo Fisher, Waltham, MA, USA) and all reactions were performed in triplicate. The relative transcript level of a gene was calculated using the 2^−ddct^ method (Livak & Schmittgen, 2001). Data were normalised to the expression of wheat *GAPDH* which was assessed in our previous study (Hao et al., 2021). Primers were designed using OLIGO 7 version software, and some primers for the *YABBY* genes were common to each set because of the highly conserved sequences in the A, B, and D subgenomes. Primers used in this study are listed in Table S1.

## Results

### Identification of YABBY in wheat

A total of 20 YABBY members were identified in the wheat genome. Among the 21 wheat chromosomes, 20 *TaYABBY* genes were unevenly distributed in 15 chromosomes according to the annotation of the wheat genome (Table 1). We designated these YABBY genes as TaYABBY1A—TaYABBY7D according to their consecutive chromosomal positions and homology relationships. All were validated using expressed sequence tags (ESTs). Among these 20 TaYABBY TFs, 18 constitute night sets, and every set contains three homoeologous genes in the A, B, and D subgenomes, respectively; two form one set with two homoeologous genes in the A and B subgenome. The deduced length of TaYABBY proteins ranged from 164 amino acids (aa) (TaYABBY2D) to 297 aa (TaYABBY1A and TaYABBY1B) with molecular weights ranging from 17.76 (TaYABBY2D) to 31.44 (TaYABBY1B) kDa. The theoretical pI ranged from 5.62 (TaYABBY2A) to 9.3 (TaYABBY6A and TaYABBY6B), and the GRAVY of each TaYABBY protein was less than zero, indicating that they are hydrophilic proteins. All the 20 YABBY genes are predicted using CELLO v.2.5 (Yu et al., 2006) and Plant-mPLoc (Chou & Shen, 2010) to be located in nuclear.

**Table 1:** Physicochemical properties of YABBY in wheat.

New name	ID	Chromosome location			Subcellular location	Number of amino acids (aa)	Molecular weight (Da)	Theoretical pI	GRAVY	EST
*TaYABBY1A*	TraesCS1A02G176300	1A	314620483	314624358	Nuclear	297	31,404.47	8.62	−0.349	22
*TaYABBY1B*	TraesCS1B02G203800	1B	367839427	367843332	Nuclear	297	31,444.45	8.62	−0.353	30
*TaYABBY1D*	TraesCS1D02G162600	1D	233307158	233311289	Nuclear	296	31,280.33	8.62	−0.328	24
*TaYABBY2A*	TraesCS2A02G197200	2A	166829162	166830134	Nuclear, Extracellular	166	17,846.33	5.62	−0.352	4
*TaYABBY2B*	TraesCS2B02G224700	2B	214534189	214535173	Nuclear Extracellular	168	18,102.65	5.64	−0.376	4
*TaYABBY2D*	TraesCS2D02G205100	2D	157017464	157019463	Nuclear Extracellular	164	17,758.29	5.92	−0.347	4
*TaYABBY3A*	TraesCS2A02G386200	2A	631967177	631969484	Nuclear	262	28,473.22	8.14	−0.423	41
*TaYABBY3B*	TraesCS2B02G403100	2B	571531403	571533593	Nuclear	268	28,855.63	6.74	−0.302	41
*TaYABBY3D*	TraesCS2D02G382700	2D	486955433	486957936	Nuclear	269	28,955.71	6.74	−0.323	41
*TaYABBY4A*	TraesCS4A02G058800	4A	51137760	51142248	Nuclear	200	22,304.67	8.98	−0.532	38
*TaYABBY4B*	TraesCS4B02G245900	4B	509033072	509037528	Nuclear	198	22,093.41	8.98	−0.552	36
*TaYABBY4D*	TraesCS4D02G245300	4D	412713684	412717920	Nuclear	200	22,318.7	8.98	−0.531	37
*TaYABBY5A*	TraesCS5A02G025900	5A	20992825	20998791	Nuclear	207	22,915.81	8.97	−0.537	24
*TaYABBY5B*	TraesCS5B02G025100	5B	24008309	24014574	Nuclear	207	22,663.48	9.13	−0.514	43
*TaYABBY5D*	TraesCS5D02G033700	5D	32297891	32303526	Nuclear	204	22,376.21	9.28	−0.5	22
*TaYABBY6A*	TraesCS5A02G371500	5A	570321614	570331718	Nuclear, Extracellular	185	20,980.93	9.3	−0.523	29
*TaYABBY6B*	TraesCS5B02G373600	5B	551331490	551338856	Nuclear Extracellular	185	20,980.93	9.3	−0.523	29
*TaYABBY7A*	TraesCS6A02G237700	6A	446900609	446903597	Nuclear	250	26,684.33	8.13	−0.196	29
*TaYABBY7B*	TraesCS6B02G266200	6B	478752450	478755692	Nuclear	250	26,652.34	8.13	−0.167	45
*TaYABBY7D*	TraesCS6D02G220400	6D	310520716	310523936	Nuclear	250	26,684.33	8.13	−0.196	45

### Collinearity analysis

**Figure 1:**
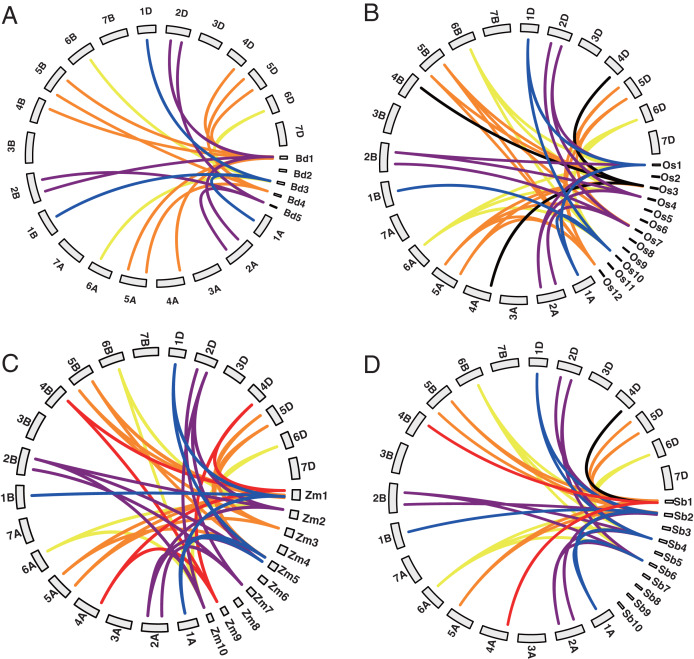
Collinearity analysis between wheat and (A) Brachypodium, (B) rice, (C) maize, and (D) sorghum. The lines represent collinearity genes.

Among the 20 *TaYABBY* genes, three segmental duplication events were constructed by four *TaYABBY* genes (Table S2). In addition, 0, 0, 7, 8, 12, and 8 orthologous were found between wheat and Arabidopsis, *Brassica napus*, *Brachypodium*, rice, maize, and sorghum respectively (Table S3 and Fig. 1). These results indicate that *TaYABBY* genes in monocot plants are closely related.

### Phylogenetic tree of wheat YABBYs

**Figure 2:**
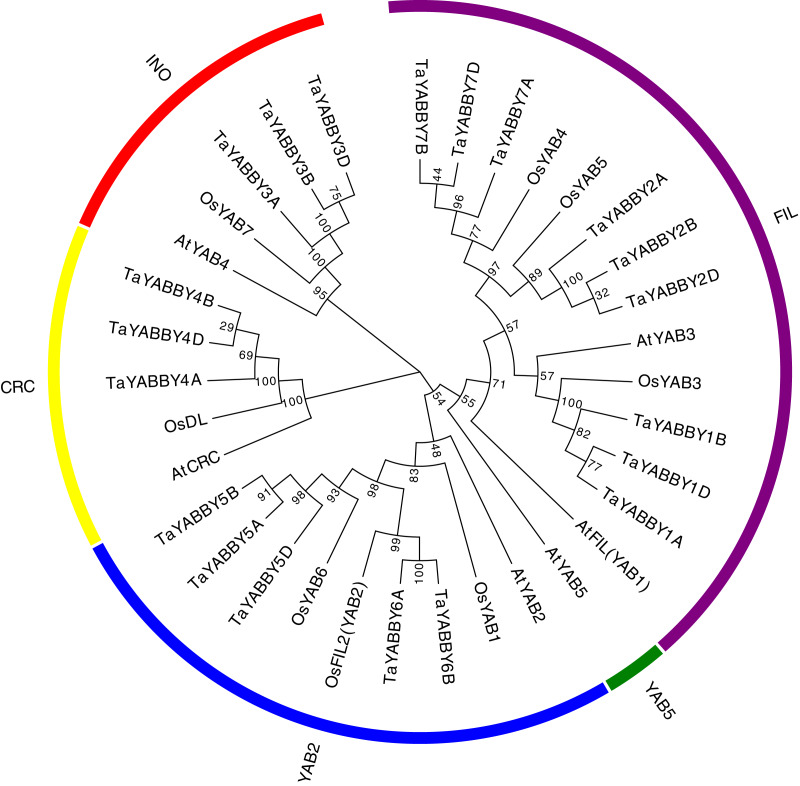
Phylogenetic relationship of wheat and rice, Arabidopsis *YABBY* genes.

To better understand the phylogenetic relationship of *YABBY* genes among rice, wheat, and its ancestor species, an NJ tree was constructed. Consistent with previous reports, these YABBY proteins can be classified into five subgroups, including 5 CRC, 5 INO, 9 YAB2, 14 FIL, and 1 YAB5 member (Fig. 2). To further evaluate the accuracy of the NJ tree, we created a tree topology using the maximum likelihood (ML) method. This tree topology was the same as that of the NJ tree in Fig. S1, indicating that the tree is suitable for further analysis. In clade YAB1, no genes of wheat or its ancestor species were included. Clade FIL is a large group, which contains night wheat YABBY members.

An unrooted NJ phylogenetic tree was also constructed in wheat YABBYs; 9, 5, 3, and 3 TaYABBYs were classified into the FIL, YAB2, INO, and CRC groups (Fig. 2A).

### Structural characteristic analysis of TaYABBY TFs

**Figure 3:**
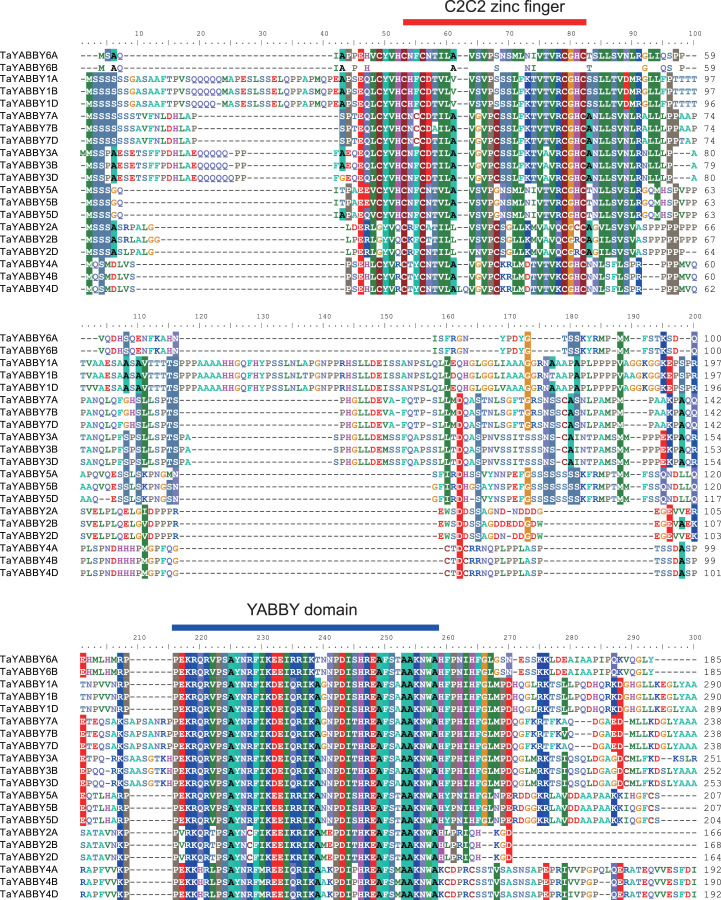
Sequence alignment of the wheat YABBY proteins. Two conserved regions were identified, including C2C2 zinc finger region in the N-terminal and YABBY domain in the C-terminal.

Two highly conserved domains were identified in all TaYABBYs, including a C2–C2 zinc finger domain at the N-terminus and a YABBY domain at the C-terminus (Fig. 3). Within the C2–C2 domain (C-X2-C-X20-C-X1-HC), the cysteine (C) and histidine (H) residues directly involved in Zn^2+^ binding are conserved. At the C-terminus, 28 conserved amino acids were 100% conserved inside the YABBY domain, including five alanine (A), three proline (P), three serine (S), three isoleucine (I), and other amino acid residues. The YABBY domain, like the HMG-box domain, has been confirmed to be associated with DNA binding (Sawa et al., 1999). In plants, the YABBY domain of Arabidopsis CRC is able to bind to the promoter regions of KCS7 and KCS15, two genes involved in the synthesis of very long chain fatty acids (Han, Yin & Xue, 2012). The YABBY domain of rice OsYABBY1 specifically binds to a GA-responsive element in the promoter of 2GA3ox2 (Dai et al., 2007), and the FIL has been confirmed to bind non-specifically to DNA *via* its YABBY domain (Kanaya, Nakajima & Okada, 2002). These results indicate that YABBY domain is the main structural domain that performs the function, and that the main amino acids play dominant roles.

**Figure 4:**
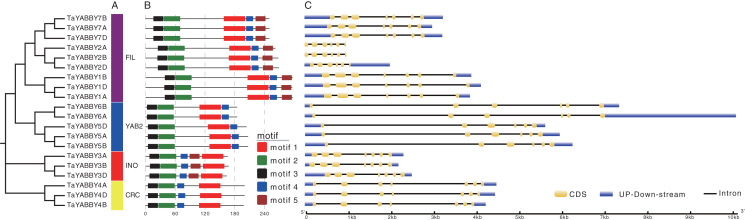
Phylogenetic, conserved motifs, and gene structures analyses of the wheat YABBY TFs. (A) Wheat YABBY TFs were classified into four clades, including FIL, YAB2, INO, and CRC. (B) Five conserved motifs were identified in TaYABBYs. (C) Gene structures of *TaYABBY* genes.

Members of the same group shared similar conserved motifs and structures. As shown in Fig. 4B, motifs were conserved within the same group; motifs 1, 2, 3, and 4 were found in all TaYABBYs, while motif 5 was only identified in FIL and INO members. Among them, motifs 1 and 4 constitute the YABBY domain, while motifs 2 and 3 form the C2–C2 domain at the N-terminus. All *TaYABBY* genes contained six or seven exons, and the phylogenesis-related genes had similar gene structures (Fig. 4C).

### Expression pattern of wheat YABBY genes

**Figure 5:**
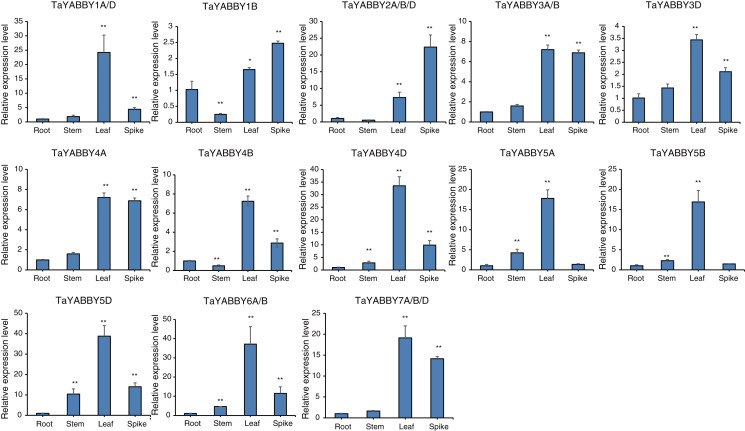
Expression patterns of *TaYABBY* genes in different tissues by qRT-PCR. The horizontal coordinates indicate the different tissues and the vertical coordinates indicate the relative expression levels. Student’s *t*-test demonstrated that statistically significant differences: *P < 0.05; **P < 0.01.

We used quantitative reverse transcription PCR (qRT-PCR) to analyse the expression patterns of *TaYABBY* genes in different tissues. The results showed that many *TaYABBY* genes were highly expressed in leaf tissues (Fig. 5), including *TaYABBY1A*/D, *4A*, *4B*, *4D*, *5A*, *5B*, *5D*, *6A/B*, and 7A/B/D. Some homoeologous genes had similar expression patterns; for example, *TaYABBY4A*, *TaYABBY4B*, and *TaYABBY4D* are highly expressed in leaf tissues; *TaYABBY7A*, *TaYABBY7B*, and *TaYABBY7D* are mainly expressed in leaf tissues.

**Figure 6:**
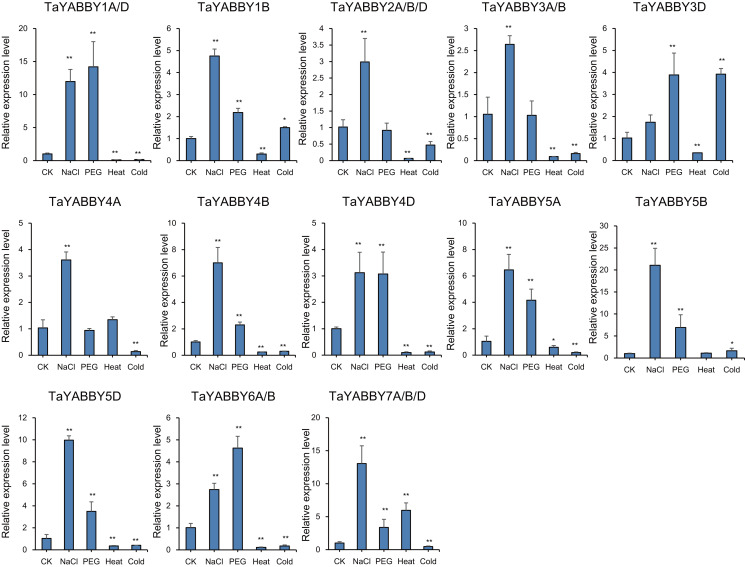
Expression patterns in *TaYABBY* genes under different abiotic stresses. The horizontal coordinates indicate different abiotic stresses and the vertical coordinates indicate the relative expression levels. Student’s *t*-test demonstrated that statistically significant differences: *P < 0.05; **P < 0.01.

**Figure 7:**
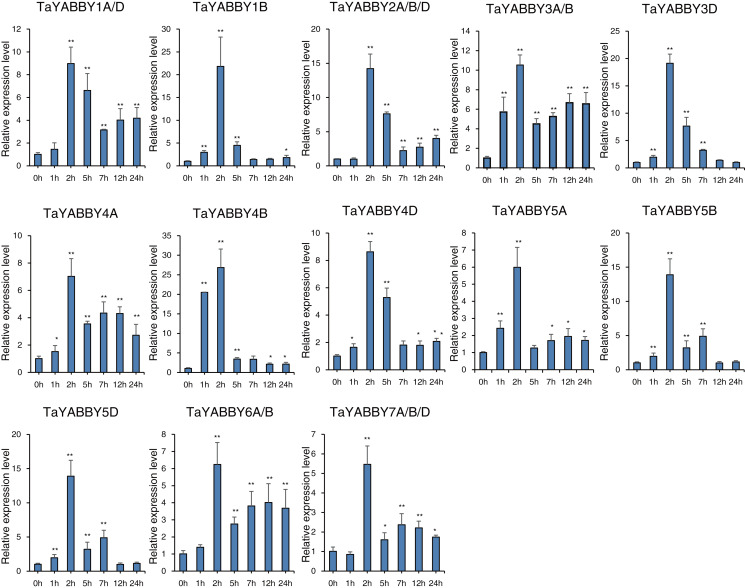
qRT-PCR analysis of *TaYABBY* genes under salt stress at different time points. The horizontal coordinates indicate the different time points and the vertical coordinates indicate the relative expression levels. Student’s *t*-test demonstrated that statistically significant differences: *P < 0.05; **P < 0.01.

We also analysed the expression patterns under different abiotic stress conditions. As shown in Fig. 6, the expression of all of them was induced by abiotic stresses; 13 and 10 were upregulated by salt and PEG treatments, respectively, while 10 and 10 were down-regulated by heat and cold treatments, respectively. Because all *TaYABBY* genes were upregulated by salt, we also analysed the expression patterns of *TaYABBY* genes under salt stress at different time points. As shown in Fig. 7, the expression levels of all genes peaked at 2 h, and then began to decline, indicating that the expression of the YABBY gene can be induced in a short period of time by salt stress. These results indicate that *TaYABBY* genes are involved in plant responses to abiotic stresses.

## Discussion

In the present study, 20 YABBY TFs were identified in wheat. There are more YABBY members in wheat than in rice (Toriba et al., 2007), Arabidopsis (Siegfried et al., 1999), soybean (Zhao et al., 2017), tomato (Huang et al., 2013), and *Moso Bamboo* (Ma et al., 2021), indicating that wheat YABBY has a more complex function. This reason is because wheat is a heterozygous polyploid. Phylogenetic analysis revealed that all TaYABBY TFs are classified into four clades: FIL, YAB2, INO, and CRC. The YAB5 clade does not exist in rice and other monocots (Toriba et al., 2007), perhaps because YABBY has undergone functional differentiation during the process of plant evolution.

A collinearity analysis showed that wheat *YABBY* genes are more closely related to those in monocot plants and have no collinearity relationship with dicotyledonous plants, further indicating that monocotyledonous and dicotyledonous species diverged functionally during evolution. Wheat YABBY TFs in the same group shared similar gene structures with each other and contained highly conserved domains, indicating that they have similar functions. Among the motifs, 1, 2, 3, and 4 were found in all wheat YABBY members, and each of them contained only six or seven exons, further indicating that they have similar functions.

Studies have shown that *YABBY* plays a role in plant growth and development, including floral organ development (Murai, 2013), leaf development (Ohmori et al., 2011), and lateral organ development (Sarojam et al., 2010). In wheat, *TaDL* (*TaYABBY4A* in this study) has been shown to regulate pistil specification (Murai, 2013); while overexpression of *TaYAB1* in *Arabidopsis* affects the formation of leaf adaxial polarity (Zhao et al., 2006); and overexpression of *TaYAB2* (*TaYABBY6A* in this study) in *Arabidopsis* causes adaxial epidermis abaxialization (Zhao et al., 2012). Compared with other studies in rice and Arabidopsis, the phylogenetically related members share conserved functions, for example, CRC members are functionally conserved in the development of floral organs (Ohmori et al., 2011; Villanueva et al., 1999; Yamaguchi et al., 2004). In this study, qRT-PCR analysis showed that all *TaYABBY* genes were highly expressed in leaf tissue, and some gene were highly expressed in spikes. It is evident that *YABBY* genes play an important role in plant growth and development.

*YABBY* genes also play a role in plant responses to abiotic stresses. For example, overexpression of pineapple *AcYABBY4* in *Arabidopsis* results in sensitivity to salt (Li & Li, 2019), and overexpression of soybean *GmYABBY10* results in sensitivity to drought, salt, and abscisic acid (ABA) (Zhao et al., 2017). In this study, expression profiles showed that the expression patterns of *TaYABBY* genes were up- and down-regulated under abiotic stresses, especially in response to salt stress. Moreover, qRT-PCR analysis showed that all *TaYABBY* genes were induced by salinity and were significantly regulated. These results suggest that *TaYABBY* genes play vital roles in plant responses to abiotic stress, especially salt stress, further indicating that the expression levels of *TaYABBY* genes are altered by abiotic stress.

## Supplemental Information

10.7717/peerj.12855/supp-1 Supplemental Information 1 Maximum Likelihood phylogenetic tree of wheat and rice, Arabidopsis *YABBY* genes.

10.7717/peerj.12855/supp-2 Supplemental Information 2 Supplemental Tables.

## Additional Information and Declarations

### Data Availability

The following information was supplied regarding data availability: The raw data are available in the Supplemental File.
