# Supplementary figures and images for: Identification and expression profiles of the YABBY transcription factors in wheat
Source: PeerJ. 2022 Feb 3;10:e12855. doi: 10.7717/peerj.12855 (PMC8818270; doi:10.7717/peerj.12855)

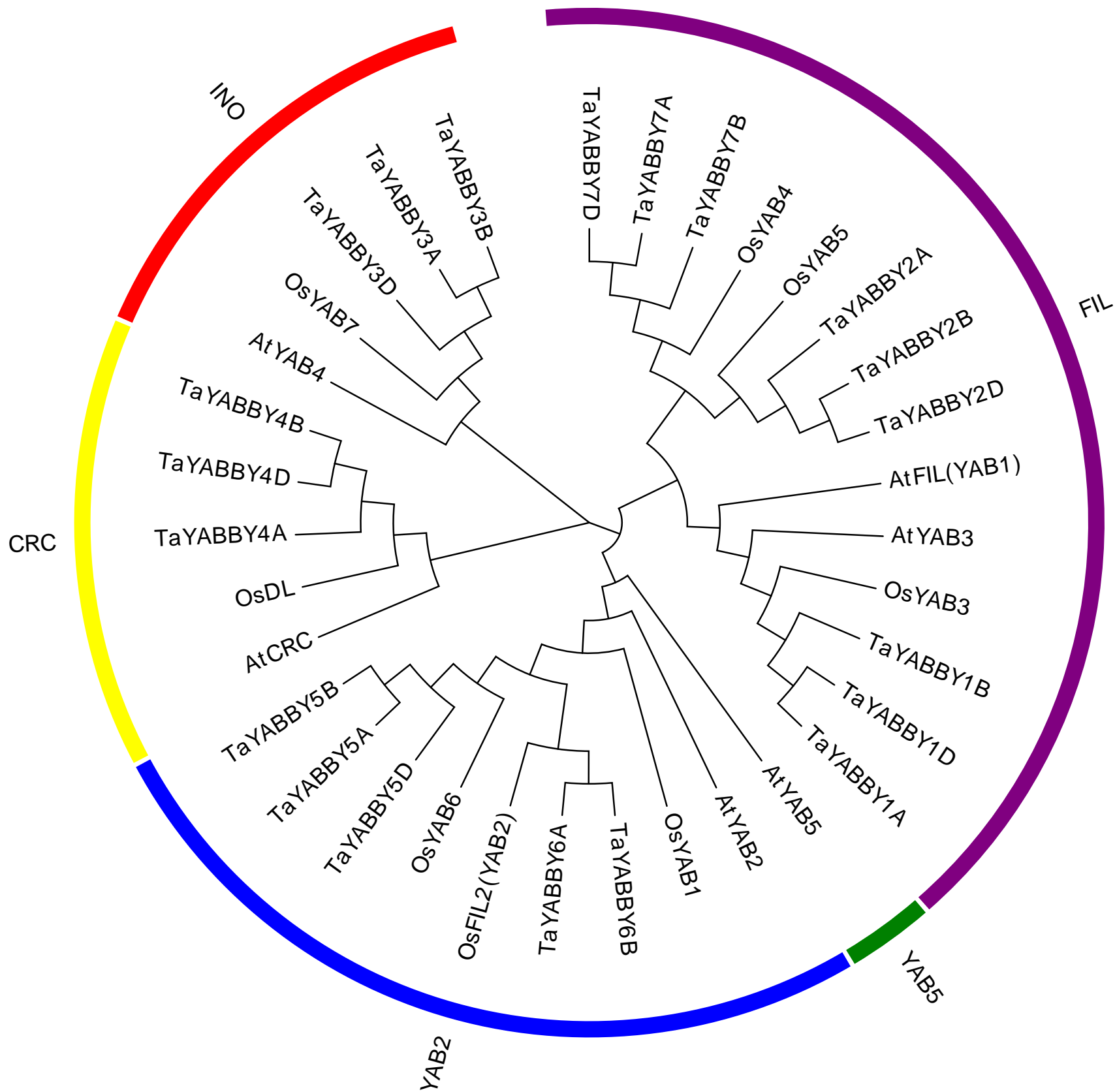

Supplement: Supplemental Information 1 [file peerj-10-12855-s001.pdf]
